# Oral Health, Caries Risk Profiles, and Oral Microbiome of Pediatric Patients with Leukemia Submitted to Chemotherapy

**DOI:** 10.1155/2021/6637503

**Published:** 2021-01-16

**Authors:** Yan Wang, Xinyi Zeng, Xue Yang, Jiajia Que, Qin Du, Qiong Zhang, Jing Zou

**Affiliations:** ^1^State Key Laboratory of Oral Diseases & National Clinical Research Center for Oral Diseases & Department of Paediatric Dentistry, West China Hospital of Stomatology, Sichuan University, Chengdu 610041, China; ^2^Department of Pediatrics, West China Second University Hospital, Sichuan University, Chengdu 610041, China; ^3^Key Laboratory of Obstetric & Gynecologic and Pediatric Diseases and Birth Defects, Ministry of Education, Sichuan University, Chengdu 610041, China; ^4^Department of Stomatology, Sichuan Provincial People's Hospital, Chengdu 610041, China

## Abstract

**Background:**

Chemotherapy is the primary treatment modality used for patients with acute lymphoblastic leukemia (ALL), but inevitably causes microbiota-related oral complications. This study is aimed at investigating the effects of chemotherapy on oral health status, caries risk, and oral microbiome in pediatric patients with ALL.

**Methods:**

Thirty-nine children with ALL receiving chemotherapy were enrolled, and a gender-, age-, dentition stage, and socioeconomic class matched healthy counterpart were recruited. Demographic information and overall health condition were obtained through the questionnaire and medical records. Oral examination was performed to assess caries and salivary status, plaque index, and other oral manifestations. Cariogram was used to assess the overall caries risk. Supragingival samples of thirteen ALL subjects and their counterparts were randomly selected to perform a 16S ribosomal RNA gene 454 pyrosequencing. Raw sequence data were screened, trimmed, and filtered using Seqcln and MOTHUR.

**Results:**

The prevalence of dental caries, gingivitis, oral mucositis, xerostomia, and candidiasis in ALL groups was higher than that of the control group (*p* < 0.05). Children with ALL demonstrated higher caries risk compared to healthy controls (HC) based upon Cariogram (*p* < 0.05). The oral microbial structure of ALL patients receiving chemotherapy is different from that of healthy controls. Oral microbiota of ALL groups showed less alpha diversity and significant differences in the composition of the oral microbiome compared to healthy controls.

**Conclusions:**

ALL patients receiving chemotherapy demonstrated compromised oral health, high caries risk, alteration of caries-related factors, and dysbiosis of oral microbiota. These findings may be of clinical importance in developing better strategies for personalized preventive management of oral diseases for pediatric children with ALL.

## 1. Introduction

Leukemia is a common malignant tumor disease in children, characterized by chromosomal abnormalities and genetic alterations involved in the differentiation and proliferation of lymphoid precursor cells [1]. ALL is the most common cancer in children with a peak incidence rate between 1 year and 4 years old. With the rapid progress in combination chemotherapy for childhood ALL, the cure rate for ALL is around 90% [2]. The Chinese Children's Leukemia Group- (CCLG-) acute lymphoblastic leukemia (ALL) 08 protocol for childhood ALL was established in 2008. And the CCLG-ALL 2008 regimen has provided a model of multiagent chemotherapy for ALL patients and achieved good therapeutic effects [3]. Methotrexate (MTX) is an essential drug in the treatment of ALL. Administration of high-dose methotrexate (HDMTX) followed by calcium folinate to “rescue” normal tissues from toxic effects is a critical component of contemporary ALL regimens [3, 4]. However, children with ALL often suffer from the toxicity of chemotherapeutic drugs such as MTX. The adverse effects of chemotherapy frequently involve the oral cavity, such as destroying oral mucosal tissues, inhibiting patients' immune function, causing dysbiosis and infection, and changing patients' eating habits and oral hygiene [5, 6]. The oral complications include oral mucositis, opportunistic infections, gingival inflammation, and bleeding, as well as xerostomia [7].

The side effects of chemotherapy impair the oral health of patients with leukemia chemotherapy and increase the susceptibility to caries [8]. Patients treated with chemotherapy were found to have a low pH and buffer ability of the saliva [9]. The changes in the quantity and quality of saliva and dietary faults make patients' oral hygiene worse. During therapy, severe pancytopenia and suppressed immune function in patients are considered further increasing the risk of dental infection [10, 11].

Caries risk refers to the possibility of individuals developing carious lesions over a certain period [12]. Assessing caries risk is an essential element in the planning of preventive and therapeutic strategies [13]. The Cariogram model proposed by Bratthall and Petersson in 2005 has higher accuracy in predicting the trend of caries development and is an ideal multifactor caries risk assessment model [13–15]. It graphically displays an individual's caries risk profile, simultaneously taking into account the interaction between different causative factors and parameters of caries [16]. Assessing the caries risk of ALL patients, especially children, and taking targeted preventive measures will help reduce the incidence of caries and improve the quality of life of children with leukemia.

The lymphoid progenitor cells of ALL patients are affected, and the side effects of chemotherapy may partially impair the immune system of the host [17–19]. The structure of the microbiome is determined by the host and environmental factors. If any of these factors are greatly disturbed, it will affect the composition of microbiome in the host, resulting in disease. Previous studies have shown that a great part of patients with ALL presented some lesions in the oral cavity during or after chemotherapy treatment such as mucositis, candidiasis, and gingivitis [20]. There is evidence indicating that oral flora is responsible for 23% to 50% of cases of septicemia in neutropenic cancer patients [21–23]. However, there is a scarcity in the literature investigated the component of oral microbiological in ALL children receiving chemotherapy. The oral cavity harbors one of the most complex microbiomes in the body [24]. The high-throughput sequencing of 16S rRNA genes is characterized by deep coverage depth and multiple output data, which can detect species with low DNA content and uncultivable taxa, providing more comprehensive information on complex microbiome [25]. It has been widely applied to the exploration of oral microbial diversity [26–28] and head-neck tumor undergone radiotherapy [29–31]. This study provided a comprehensive comparison of oral microbiological components in ALL patients receiving chemotherapy and HC for each leukemia child using 16S ribosomal RNA gene 454 pyrosequencing, which helps to better manage related oral and systemic complications.

In general, this study chose ALL pediatric patients receiving chemotherapy as the research object to investigate the oral health status, caries risk, and oral microbiome composition comparing the HC matched for each leukemia child. The results of this study may help guide oral hygiene practices and clinic oral operation for ALL populations and take targeted preventive measures to improve the quality of life.

## 2. Methods

### 2.1. Study Population

This matched-case control study was approved by the Ethics Committee of State Key Laboratory of Oral Diseases, Sichuan University, Chengdu, China. The informed consent was obtained from the parents or guardians before the investigation. The leukemia children were patients at the Department of Pediatric Hematology and Oncology. Eligible participants were those diagnosed with acute lymphoblastic leukemia that had taken the CCLG-ALL 2008 chemotherapy regimen for a minimum of one year. Exclusion criteria were as follows: without receiving stem cell transplantation, patients older than 18 years old, systemic diseases other than acute lymphoblastic leukemia, and acute oral infection such as dental abscess. For the control group, “social twins” were recruited from the Department of Pediatric Dentistry that matched age, gender, and caries status. The detailed inclusion/exclusion criteria are listed in Table 1.

### 2.2. Questionnaire

Questionnaires were distributed to all subjects and completed on the day of oral examination. The content of the questionnaire included the basic information of the sample, the type and frequency of diet, oral hygiene habits, fluoride application, and systemic diseases. For the leukemia group, current and prior chemotherapy protocols and duration of chemotherapy were inquired and further confirmed by checking the corresponding medical records.

### 2.3. Clinical Examination

The examination was carried out by two of the authors (Que JJ and Wang Y). Before the start of the investigation, the examiner was calibrated with an experienced pediatric dentist (Zou J). A probe and mirror under optimal light conditions were available for all dental examinations.

Caries status was recorded according to the World Health Organization (WHO) criteria [32]. These included the number of tooth surfaces decayed, the number of teeth missed due to caries, and the number of surfaces restored after decay. For the primary teeth, the caries parameter was registered as decayed missing filled teeth (dmft); for the permanent teeth, it was recorded as Decayed Missing Filled Teeth (DMFT).

Oral hygiene status was registered according to the plaque index system developed by Silness and Loe [33]. It was assessed on the buccal, lingual, mesial, and distal surfaces of the test teeth. Each surface was scored by the same examiner, and the percent of each score was calculated by dividing the number of surfaces presenting a certain score by the total number of surfaces examined. If the tooth was missing, the adjacent tooth was checked.

### 2.4. Salivary Analyses

The tests were done at least 1 hour after tooth brushing, eating, or drinking. Because the saliva secretion fluctuated at different time points during the day, all tests were accomplished between 9 and 11 a.m. to make all subjects comparable.

#### 2.4.1. Salivary Flow Rate

A paraffin pellet was given to the subject to chew for 30 seconds and then spit out the accumulated saliva. The subject then continues to chew the paraffin for 5 minutes with the accumulated saliva continuously collected into a measuring cup. The secretion rate was calculated by dividing the amount of saliva by the time used.

#### 2.4.2. Salivary Buffer Capacity

A drop of the collected saliva was added to the test pad of Dentobuff® Strip (Orion Diagnostica, Espoo, Finland) for measuring buffer capacity according to the manufacturer's instructions, expressed as low (yellow), medium (middle), and high (blue).

#### 2.4.3. Streptococcus Mutans Count

*Streptococcus mutans* count was estimated by using the Dentobuff® SM test. The test strip was gently turned 10 times on the back of the subject's tongue, placed in the medium, and cultured at 37°C for 48 hours after counting the *Streptococcus mutans* and reading the results.

#### 2.4.4. Cariogram

Data were collected according to the Cariogram operation manual and entered into the Cariogram model for scoring as previously described [14]. The items include caries experience, related diseases, diet contents, diet frequency, plaque amount, *Streptococcus mutans*, fluoride program, saliva secretion, and saliva buffer capacity. Among these, related diseases refer to general diseases or conditions associated with dental caries such as Sjogren's syndrome, continuous use of drugs that affect saliva secretion function, and head-neck tumor chemotherapy. According to its built-in formula, the program presents a pie diagram which is composed of 5 parts of different colors:

- The dark blue sector “Diet” is based on a combination of diet contents and diet frequency
- The red sector “Bacteria” is based on a combination of the amount of plaque and *Streptococcus mutans*
- The light blue sector “susceptibility” is based on a combination of fluoride program, saliva secretion, and saliva buffer capacity
- The yellow sector “Circumstances” is based on a combination of caries experience and related diseases
- The green sector shows an estimation of the “Chance of avoiding caries”

According to the “Chance of avoiding caries” (the proportion of green sector area) in the results, the subjects can be divided into the high-risk group (<40%), medium-risk group (40%~60%), and low-risk group (>60%).

### 2.5. Oral Microbiome Sample Collection

Supragingival samples of thirteen ALL subjects and their counterparts were randomly selected. Microbial samples were collected at the same time of day, approximately two hours after breakfast, using the method mentioned in the Manual of Procedures for Human Microbiome Project with minor modifications. Briefly, the sampling sites, the teeth in the upper right and lower left quadrants or upper left and lower right quadrants, were isolated with cotton rolls and dried before sampling. A sterile Gracey curette was used to collect a pooled supragingival plaque sample from the mesial surfaces of each of these teeth in turn. The collected plaque samples were released from the curette by agitation in 700 ml of TE buffer (10 mM Tris-Cl [pH 7.5] and 1 mM EDTA). The microbial samples were immediately transported on ice to the laboratory and stored at 280 *μ*C until further DNA extraction and pyrosequencing analysis.

### 2.6. DNA Extraction and Pyrosequencing

Bacterial DNA was extracted using QIAamp DNA Micro Kit (QIAGEN, Hilden, Germany) according to the manufacturer's instructions with minor modification. Briefly, 30 ml lysozyme solution (50 mg/ml) was added to the mixture to increase the yield of bacterial DNA from Gram-positive bacteria. The amount of DNA extracted per sample was determined using Quant-iT PicoGreen dsDNA Assay Kit. The 16S rRNA hypervariable V1–V3 region was amplified using FastStart High Fidelity PCR System, dNTP Pack (Roche Applied Science) according to previous descriptions [34]. Obtained sequences from the pyrosequencer were also analyzed according to the previous study [34]. To optimize the raw sequences, the Seqcln and MOTHUR (version 1.30.1) were applied to screen, trim, and filter the data. Qualified sequences were submitted to the SILVA database (SILVA 111) for taxonomic alignment. Community richness (ACE, Chao1) and diversity index (Shannon index and Simpson index) were determined by the MOTHUR program at 97% similarity level. Metastats was used to compare the relative abundance of each taxon at different taxonomic levels between ALL patients and healthy controls [35].

### 2.7. Statistical Analyses

Data were entered into SPSS 24.0 (SPSS Inc., Chicago, IL, USA). For the quantitative data and the nonnormal distribution of quantitative data, the independent samples Kruskal-Wallis test was used to assess the difference in caries risk between the ALL group and HC group, and the paired rank-sum test and Fisher's exact test were used to compare the caries risk factors of two groups. The statistical significance of the oral microbiome was determined by the nonparametric Mann-Whitney *U* test for independent samples. The *p* value threshold was set at 0.05, and the *q* value threshold at 0.5.

## 3. Results

### 3.1. Characteristics of Study Population

We enrolled 39 acute lymphoblastic leukemia patients and 39 healthy counterparts. There were 23 males and 16 females in the ALL group, with an average age of 6.9 ± 3.92 years. The age, gender, socioeconomic class, and dentition stage of the control group matched the ALL group. The characteristics of the investigated subjects are shown in Table 2.

### 3.2. Oral Health Status

The prevalence rate of different oral diseases in acute lymphoblastic leukemia and healthy controls is presented in Table 3. The prevalence of tested oral diseases in the ALL group was showed higher than that of HC group. Among which, the number of cases of oral mucositis and candidiasis in the HC group was 0, while that in the ALL group was 6 and 1, respectively, accounting for 15.4% and 2.6%. The incidence of dental caries and gingivitis in the ALL group was very high, accounting for 69.2% and 38.5%, respectively. In terms of xerostomia, the prevalence of the ALL group was 25.6%, also higher than 2.1% in the HC group.

### 3.3. Caries Risk

We compared the following caries-related factors: caries experience, related diseases, diet content, diet frequency, plaque amount, *Streptococcus mutans*, fluoride program, saliva secretion, and saliva buffering capacity (Table 4). Compared with healthy controls, we found significantly higher sugar consumption of diet intake as well as a higher frequency in ALL group (*p* = 0.002). The average plaque index was 1.47 ± 0.70 in ALL group, more than that of HC group with 1.13 ± 0.33 (*p* = 0.008). And the average stimulated salivary secretion rate was 0.78 ± 0.41 in ALL group, less than 1.01 ± 0.40 in HC group (*p* = 0.017). Children with ALL received less fluoride program than healthy controls (*p* = 0.006). No statistical difference was found concerning salivary buffer capacity and counts of *Streptococcus mutants* in the whole saliva (*p* > 0.05).

**Figure 1:**
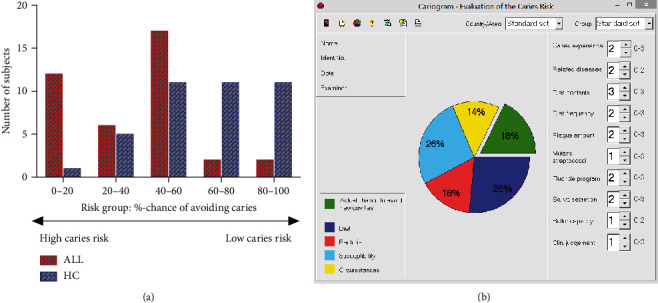
Distribution of Cariogram risk categories in acute lymphoblastic leukemia and healthy subjects. (a) Risk grouping was based on percent chance of avoiding caries. (b) Example of a Cariogram indicating high caries risk with the “chance of avoiding caries (new cavities)” estimated to18%. ALL: patients with acute lymphoblastic leukemia; HC: healthy controls.

The Cariogram was applied to evaluate the caries risk, and the distribution of Cariogram risk categories in ALL and healthy controls is shown in Figure 1(a). The adolescents were divided into 5 risk subgroups according to the: (1) 0–20% (high caries risk), (2) 21–40%, (3) 41–60%, (4) 61–80%, and (5) 81–100% (low caries risk). The median of the “chance of avoiding caries” was 41.76% in the ALL group and 64.54% in the HC group. A significant difference was found between the ALL and HC groups in the caries risk (*p* < 0.001). The individual Cariogram sectors contributing to the higher risk profiles for leukemia adolescents were “susceptibility” (*Streptococcus mutants*, fluoride, and saliva buffer capacity) and “diet” (*p* < 0.01). An example of a Cariogram is shown in Figure 1(b).

### 3.4. Oral Microbiome

**Figure 2:**
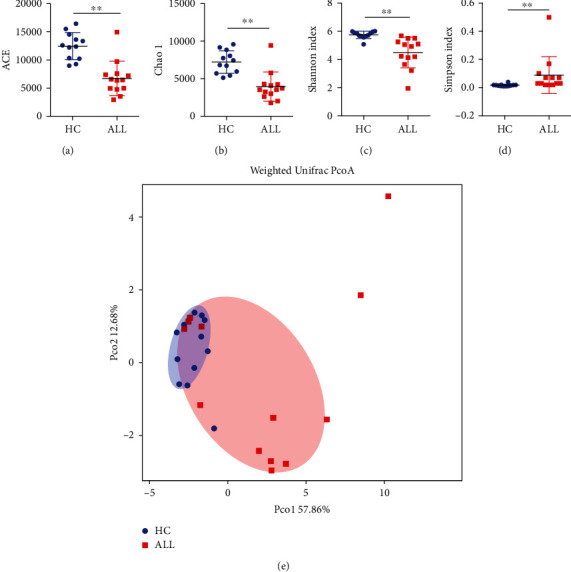
Alpha diversity and weighted Unifrac PCoA analysis of acute lymphoblastic leukemia and healthy subjects. (a) ACE. (b) Chao1. (c) Shannon index. (d) Simpson index. ^∗∗^*p* < 0.01. (e) Weighted Unifrac PCoA analysis. ALL: patients with acute lymphoblastic leukemia; HC: healthy controls.

In this study, 13 ALL subjects and their counterparts were randomly selected, from which qualified sequence reads were obtained and used for analysis. The sequencing data were analyzed with MOTHUR for alpha diversity analysis, including ACE, Chao1, Shannon, and Simpson (Figures 2(a)–2(d)). Alpha diversity analysis, including Chao, ACE, Shannon, and Simpson, revealed a statistically significant lower estimate of richness for ALL patients receiving chemotherapy compared with healthy children (*p* < 0.01). The higher alpha diversity of the HC group may be related to the uniform distribution of different species of healthy controls.

To compare the oral microbial structure of ALL patients and healthy controls, principal coordinate analysis (PCoA) based on the weighted UniFrac metric was performed. A segregation trend for ALL patients and healthy controls was observed, especially by the principal coordinate Pco1 (Figure 2(e)), indicating that the oral microbial structure of ALL patients receiving chemotherapy is distinct from that of healthy controls.

**Figure 3:**
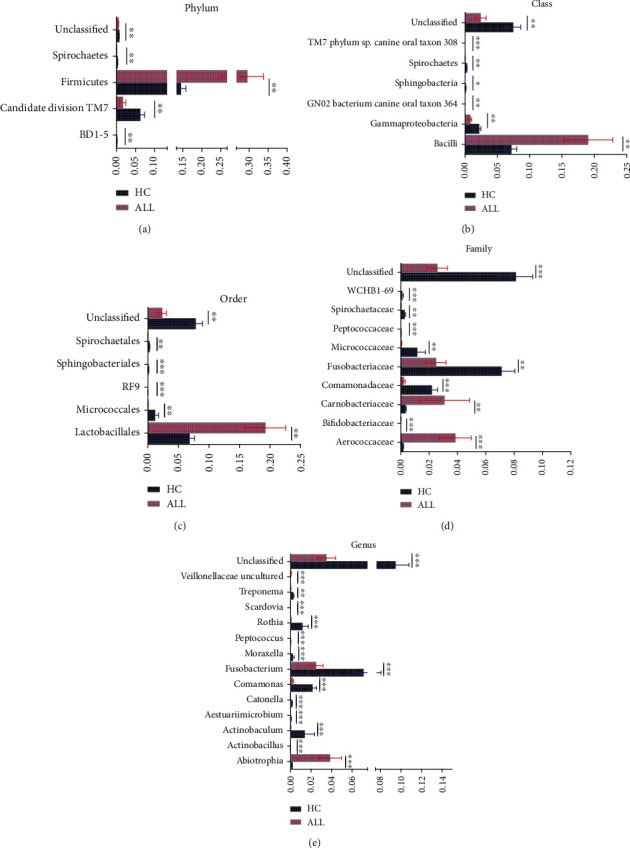
Differential relative abundance of bacterial taxonomy profiles of acute lymphoblastic leukemia and healthy subjects based on Metastats analysis. (a) Phylum level, (b) class level, (c) order level, (d) family level, and (e) genus level. ^∗^*p* < 0.05, ^∗∗^*p* < 0.01, and ^∗∗∗^*p* < 0.001.

Comparisons of oral microbiota between ALL patients and healthy controls at each of the taxonomical levels of phylum, class, order, family, and genus were performed based on meta-analysis. Statistical differences in the relative abundance of phylum, class, order, family, and genus are shown in Figures 3(a)–3(e). A total of 12 phyla were identified in oral microbiota of ALL patients and healthy children in our dataset, which were dominated by six major phyla, including *Firmicutes*, *Proteobacteria*, *Fusobacteria*, *Actinobacteria*, *Bacterioidetes*, and *Candidate division TM7*. Particularly, notable differences in abundance between ALL patients and healthy controls were found for the two phyla, i.e., *Firmicutes* (*p* = 0.002) and *Candidate division TM7* (*p* = 0.006). At a further taxonomic level, there were 6 classes, 5 orders, 9 families, and 13 genera that were identified to have statistical differences in the oral cavity of ALL patients and healthy controls (*p* < 0.05), and the detailed *p* values for each specific taxon are presented in Figure 3.

## 4. Discussion

This study comprehensively compared the effects of chemotherapy on oral health status, caries risk, and oral microbial composition of ALL and healthy counterparts. To assess the oral health of ALL children, the current study evaluated their degree of caries and the prevalence rate of different oral diseases. And the caries risk was estimated with Cariogram by measuring nine factors of relevance to caries. Moreover, the high-throughput sequencing of 16S rRNA genes was applied to further explore and analyze the oral microbiome of the two groups. ALL patients receiving chemotherapy showed a high prevalence of oral complications and caries risk, compromised oral health, and dysbiosis of oral microbiota. While there have been several studies investigating the oral health status or microbiota in leukemia patients on chemotherapy, few have conducted a comprehensive analysis of all three [19, 36]. These findings may help to better implement a preventive oral health regimen and minimize the risk of associated oral complications, thereby improving the quality of life of patients with ALL.

Children affected by leukemia receive various forms of treatment including chemotherapeutic agents and stem cell transplants. Methotrexate as a chemotherapeutic drug can produce direct toxicity and affect the oral mucosa through the systemic circulation. Thus, chemotherapy could affect the oral health status of ALL patients, manifested by a higher prevalence of five common oral manifestations than healthy counterparts. In our study, oral disease with the highest prevalence rate in children with ALL was dental caries, accounting for 69.2%. This may be due to the toxic effects of methotrexate and its adverse effects on oral hygiene. In the study by Torres et al., gingivitis is a common oral manifestation of chemotherapeutic drugs, affecting 91.84% of the samples [37]. Similar results were obtained in this study, which may be related to poor oral hygiene caused by using chemotherapeutic drugs. Candidiasis, a fungal opportunistic infection, is also common among children with ALL in previous studies [37–39]. The presence of candidiasis could have been caused by the patients' low immunity, associated with exposure to the virus. The most frequent oral complications were mucositis, candidiasis, periodontitis, and gingivitis according to a systematic review, and high-dose chemotherapy drugs can also lead to xerostomia [20, 40].

Besides, further research on the caries of ALL patients found that the application of HDMTX affected caries-related factors, manifested by changes in content and frequency of diet, increased dental plaque, and reduced salivary flow rate compared with health counterparts, which also promoted the occurrence of the abovementioned oral diseases. Chemotherapy will cause a decrease in saliva volume and saliva flow rate in patients with ALL and manifest as xerostomia in severe cases [41]. Hong et al. considered that leukemia patients tend to consume more high-energy foods and drink sugar-rich beverages to relieve oral dryness [11]. Due to nausea and vomiting caused by chemotherapy drugs, children with ALL have a small food intake, so the number of meals has increased significantly. The high sugar consumption and increased frequency of eating are not conducive to the oral hygiene of patients, promoting the development of caries and increasing the risk of caries in children with ALL. It can be observed that the application of chemotherapy seriously damages the oral health status of children with ALL in comparison with counterparts.

As a multifactorial infectious disease, caries should be evaluated from multiple factors so that the prediction has accuracy and validity. The Cariogram, an interactive computer-based caries risk assessment program, has developed rapidly in the past years, with many related studies and high evaluations. It has been used to evaluate the caries risk of various populations, including normal populations of different ages [12, 42], patients undergoing oral treatment [43, 44], and patients with systemic diseases [45]. Agouropoulos et al. compared the validity of three caries risk assessment tools and concluded that Cariogram displayed a higher validity in predicting caries increments [46]. In this study, children with ALL revealed a lower percentage chance of avoiding caries, of which 30.77% of children were assessed as extremely high risk of dental caries (0-20% chance of avoiding caries). Therefore, it is necessary to evaluate the caries risk of ALL children during the chemotherapy period and take oral health measures for high-risk children, which can help reduce the incidence of caries in ALL children and improve their quality of life.

In the present study, we sampled supragingival plaque in children with ALL and their healthy counterparts and analyzed its microbial composition via 16S-based 454 pyrosequencing. ALL children receiving chemotherapy had a lower richness and less diversity of oral microbiota compared with healthy counterparts, which pointed to the dysbiosis of oral microbiota in ALL patients. Moreover, we reported altered structure and composition of oral microbiota in ALL patients. As we all know, the health of the host is closely related to the microbiome, and the host immune system plays an important role in maintaining the balance of the microbiome. A study on patients with acute myeloid leukemia (AML) undergoing induction chemotherapy indicated a high degree of intrapatient temporal instability of oral microbial diversity and that increased variability was correlated with adverse clinical outcomes [47]. And a study by Hong et al. demonstrated that chemotherapy-induced oral mucositis is associated with detrimental bacterial dysbiosis [48]. ALL patients may suffer from impaired host immunity due to disorders of lymphoid progenitor cells (the main part of the body's immune system), and the side effects of methotrexate exacerbate this process, leading to disruption of the oral microbiota [34]. Moreover, the application of chemotherapy seriously damaged the oral health status of children with ALL, which also promoted the dysbiosis of oral microbiota.

We studied the differential relative abundance of bacterial taxonomy profiles of children with ALL and healthy counterparts. The results showed notable differences from the phylum down to the genus level in abundance between the two groups (*p* < 0.05). The *Firmicutes* phylum, *Bacilli* class, *Lactobacillales* order, *Carnobacteriaceae* and *Aerococcaceae* families, and *Abiotrophia* genus are much more abundant in the supragingival plaque of ALL patients than healthy counterparts. At the genus level, the leukemia-depleted genera included *Fusobacteria*, *Comamonas*, *Actinobacteria*, and *Rothia*, while only *Abiotrophia* was significant leukemia-enriched genera. Hong et al. also studied the association of chemotherapy-induced oral mucositis and oral microbiome, founding the bacteriome depletion of common health-associated commensals from the genera *Streptococcus*, *Actinomyces*, *Gemella*, *Granulicatella*, and *Veillonella* and enrichment of Gram-negative bacteria such as *Fusobacterium nucleatum* and *Prevotella oris* [48]. Although the exact mechanism of the interaction between infectious diseases and microbiota has not been clarified, the study of oral microbiota in ALL patients can provide the opportunity for identifying potential infectious diseases. Hence, preventing the dysbiosis of the oral microbiota might be a promising measure for decreasing the risk of associated infectious complications in children with ALL. And further precise experimental techniques and cohort studies are needed to elucidate the exact relationship between the two.

## 5. Conclusions

ALL patients receiving chemotherapy demonstrated compromised oral health, high caries risk, alteration of caries-related factors, and dysbiosis of oral microbiota compared to healthy counterparts. These findings may be of clinical importance in developing better strategies for personalized preventive management of oral diseases for pediatric children with ALL, thereby improving their quality of life.

## Figures and Tables

**Table 1:** Inclusion and exclusion criteria.

	Inclusion criteria	Exclusion criteria
ALL group	(i) Diagnosed with acute lymphoblastic leukemia based on bone marrow samples (ii) Receiving CCLG-ALL 2008 chemotherapy regimen (iii) No more than eighteen years old (iv) Free of other systemic diseases (v) Written informed consent	(i) Receiving antibiotics within 3 months before the study (ii) Local antimicrobial treatment within 2 weeks (iii) Without receiving stem cell transplantation
Healthy control group	(i) Age and socioeconomic status comparable with ALL patients (ii) Free of systemic diseases (iii) Written informed consent	(i) Systemic disorders that cause oral mucosal lesions or periodontal diseases (ii) Receiving antibiotics within 3 months before the study (iii) Local antimicrobial treatment within 2 weeks

ALL: patients with acute lymphoblastic leukemia.

**Table 2:** Sociodemographic characteristics of the investigated subjects.

Variables	Characteristics	HC (*n* = 39)	ALL (*n* = 39)	*p* value
Age (years)	Mean ± SD	7.00 ± 3.02	6.95 ± 3.92	0.950^#^
Dentition stage	Primary	17	18	0.858^∗^
	Mixed	16	15	
	Permanent	6	6	
Gender	Male	23	23	1.000^∗^
	Female	16	16	
Socioeconomic class	More than 2000¥ per month	10	8	0.646^∗^
	1000~2000¥ per month	18	19	
	Less than 1000¥ per month	11	12	

ALL: patients with acute lymphoblastic leukemia; HC: healthy controls. ^#^Unpaired *t* test was used. ^∗^Independent sample nonparametric Mann-Whitney *U* test was used.

**Table 3:** Prevalence of oral diseases.

Variables	HC (*n* = 39)		ALL (*n* = 39)	
	*n* ^b^	%	*n* ^b^	%
Dental caries	19	48.7	27	69.2
Gingivitis	3	7.7	15	38.5
Oral mucositis	0	0	6	15.4
Xerostomia^a^	1	2.1	10	25.6
Candidiasis	0	0	1	2.6

ALL: patients with acute lymphoblastic leukemia; HC: healthy controls. ^a^Stimulated saliva secretion rate < 0.5 ml/min. ^b^There existed subjects with no or more than two oral diseases.

**Table 4:** Caries-related factors in children with acute lymphoblastic leukemia and healthy controls.

Variables	Cut-off value	HC (*n* = 39)	ALL (*n* = 39)	*p* value
Diet content	Very low sugar consumption	18	6	0.002^∗^
	Low sugar consumption	10	10	
	Moderate sugar consumption	8	17	
	High sugar consumption	3	6	
Diet frequency	<3/day	3	1	0.003^∗^
	3~5/day	32	23	
	>5/day	4	15	
Plaque index	Mean ± SD	1.13 ± 0.33	1.47 ± 0.70	0.008^#^
Streptococcus mutants	<104 CFU/ml	16	22	0.146^∗^
	10^4^~105 CFU/ml	17	14	
	10^5^~106 CFU/ml	3	1	
	>10^6^ CFU/ml	3	2	
Fluoride program	0	7	1	0.006^∗^
	1	10	4	
	2	14	20	
	3	8	14	
Stimulated saliva secretion rate	Ml/min, mean ± SD	0.78 ± 0.41	1.01 ± 0.40	0.017^#^
Salivary buffer capacity	Low	0	0	0.190^∗^
	Medium	7	12	
	High	32	27	

ALL: patients with acute lymphoblastic leukemia; HC: healthy controls. ^#^Unpaired *t* test was used. ^∗^Independent sample nonparametric Mann-Whitney *U* test was used.

## Data Availability

All data generated and analyzed in this study are included within the article or available from the corresponding author on reasonable request.

## Supplementary Materials

Supplementary Materials The detailed treatment regimens of the acute lymphoblastic leukemia patients are available in the supplementary material.
