# Supplementary material for: Oral Health, Caries Risk Profiles, and Oral Microbiome of Pediatric Patients with Leukemia Submitted to Chemotherapy
Source: Biomed Res Int. 2021 Jan 16;2021:6637503. doi: 10.1155/2021/6637503 (PMC7834790; doi:10.1155/2021/6637503)
Supplement: Supplementary Materials — The detailed treatment regimens of the acute lymphoblastic leukemia patients are available in the supplementary material. [file 6637503.f1.docx]

**Supplementary Material**

All the pediatric patients with ALL included in this study were treated with The Chinese Children's Leukemia Group (CCLG)-acute lymphoblastic leukemia (ALL) 08 regimen for chemotherapy [1]. According to the risk set by CCLG-ALL 2008 regimen, they were divided into groups and treated. Among the 39 pediatric patients in the group, 24 were at low risk, 10 at medium risk and 5 at high risk. The detailed treatment regimens of the included acute lymphoblastic leukemia patients are listed below.

Table S1 Treatment regimen of the included acute lymphoblastic leukemia patients.

| Patients | induced remission therapy | Early intensive therapy | Consolidating therapy | Delayed intensive therapy | Maintenance therapy | Risk |
| --- | --- | --- | --- | --- | --- | --- |
| ALL_1 | VDLD (DNR×2) | CAM | HD-MTX 2g/m^2^×4 | VDLD+ CAM | 6-MP+HTX/VD＋  intrathecal injection | Low |
| ALL_2 | VDLD (DNR×2) | CAM | HD-MTX 2g/m^2^×4 | VDLD+ CAM | 6-MP+HTX/VD＋  intrathecal injection | Low |
| ALL_3 | VDLD (DNR×2) | CAM | HD-MTX 2g/m^2^×4 | VDLD+ CAM | 6-MP+HTX/VD＋  intrathecal injection | Low |
| ALL_4 | VDLD (DNR×2) | CAM | HD-MTX 2g/m^2^×4 | VDLD+ CAM | 6-MP+HTX/VD＋  intrathecal injection | Low |
| ALL_5 | VDLD (DNR×2) | CAM | HD-MTX 2g/m^2^×4 | VDLD+ CAM | 6-MP+HTX/VD＋  intrathecal injection | Low |
| ALL_6 | VDLD (DNR×2) | CAM | HD-MTX 2g/m^2^×4 | VDLD+ CAM | 6-MP+HTX/VD＋  intrathecal injection | Low |
| ALL_7 | VDLD (DNR×2) | CAM | HD-MTX 2g/m^2^×4 | VDLD+ CAM | 6-MP+HTX/VD＋  intrathecal injection | Low |
| ALL_8 | VDLD (DNR×2) | CAM | HD-MTX 2g/m^2^×4 | VDLD+ CAM | 6-MP+HTX/VD＋  intrathecal injection | Low |
| ALL_9 | VDLD (DNR×2) | CAM | HD-MTX 2g/m^2^×4 | VDLD+ CAM | 6-MP+HTX/VD＋  intrathecal injection | Low |
| ALL_10 | VDLD (DNR×2) | CAM | HD-MTX 2g/m^2^×4 | VDLD+ CAM | 6-MP+HTX/VD＋  intrathecal injection | Low |
| ALL_11 | VDLD (DNR×2) | CAM | HD-MTX 2g/m^2^×4 | VDLD+ CAM | 6-MP+HTX/VD＋  intrathecal injection | Low |
| ALL_12 | VDLD (DNR×2) | CAM | HD-MTX 2g/m^2^×4 | VDLD+ CAM | 6-MP+HTX/VD＋  intrathecal injection | Low |
| ALL_13 | VDLD (DNR×2) | CAM | HD-MTX 2g/m^2^×4 | VDLD+ CAM | 6-MP+HTX/VD＋  intrathecal injection | Low |
| ALL_14 | VDLD (DNR×2) | CAM | HD-MTX 2g/m^2^×4 | VDLD+ CAM | 6-MP+HTX/VD＋  intrathecal injection | Low |
| ALL_15 | VDLD (DNR×2) | CAM | HD-MTX 2g/m^2^×4 | VDLD+ CAM | 6-MP+HTX/VD＋  intrathecal injection | Low |
| ALL_16 | VDLD (DNR×2) | CAM | HD-MTX 2g/m^2^×4 | VDLD+ CAM | 6-MP+HTX/VD＋  intrathecal injection | Low |
| ALL_17 | VDLD (DNR×2) | CAM | HD-MTX 2g/m^2^×4 | VDLD+ CAM | 6-MP+HTX/VD＋  intrathecal injection | Low |
| ALL_18 | VDLD (DNR×2) | CAM | HD-MTX 2g/m^2^×4 | VDLD+ CAM | 6-MP+HTX/VD＋  intrathecal injection | Low |
| ALL_19 | VDLD (DNR×2) | CAM | HD-MTX 2g/m^2^×4 | VDLD+ CAM | 6-MP+HTX/VD＋  intrathecal injection | Low |
| ALL_20 | VDLD (DNR×2) | CAM | HD-MTX 2g/m^2^×4 | VDLD+ CAM | 6-MP+HTX/VD＋  intrathecal injection | Low |
| ALL_21 | VDLD (DNR×2) | CAM | HD-MTX 2g/m^2^×4 | VDLD+ CAM | 6-MP+HTX/VD＋  intrathecal injection | Low |
| ALL_22 | VDLD (DNR×2) | CAM | HD-MTX 2g/m^2^×4 | VDLD+ CAM | 6-MP+HTX/VD＋  intrathecal injection | Low |
| ALL_23 | VDLD (DNR×2) | CAM | HD-MTX 2g/m^2^×4 | VDLD+ CAM | 6-MP+HTX/VD＋  intrathecal injection | Low |
| ALL_24 | VDLD (DNR×2) | CAM | HD-MTX 2g/m^2^×4 | VDLD+ CAM | 6-MP+HTX/VD＋  intrathecal injection | Low |
| ALL_25 | VDLD (DNR×4) | CAM×2 | HD-MTX 5g/m^2^×4 | VDLD+ CAM | 6-MP+MTX/VD+ intrathecal injection | Medium |
| ALL_26 | VDLD (DNR×4) | CAM×2 | HD-MTX 5g/m^2^×4 | VDLD+ CAM | 6-MP+MTX/VD+ intrathecal injection | Medium |
| ALL_27 | VDLD (DNR×4) | CAM×2 | HD-MTX 5g/m^2^×4 | VDLD+ CAM | 6-MP+MTX/VD+ intrathecal injection | Medium |
| ALL_28 | VDLD (DNR×4) | CAM×2 | HD-MTX 5g/m^2^×4 | VDLD+ CAM | 6-MP+MTX/VD+ intrathecal injection | Medium |
| ALL_29 | VDLD (DNR×4) | CAM×2 | HD-MTX 5g/m^2^×4 | VDLD+ CAM | 6-MP+MTX/VD+ intrathecal injection | Medium |
| ALL_30 | VDLD (DNR×4) | CAM×2 | HD-MTX 5g/m^2^×4 | VDLD+ CAM | 6-MP+MTX/VD+ intrathecal injection | Medium |
| ALL_31 | VDLD (DNR×4) | CAM×2 | HD-MTX 5g/m^2^×4 | VDLD+ CAM | 6-MP+MTX/VD+ intrathecal injection | Medium |
| ALL_32 | VDLD (DNR×4) | CAM×2 | HD-MTX 5g/m^2^×4 | VDLD+ CAM | 6-MP+MTX/VD+ intrathecal injection | Medium |
| ALL_33 | VDLD (DNR×4) | CAM×2 | HD-MTX 5g/m^2^×4 | VDLD+ CAM | 6-MP+MTX/VD+ intrathecal injection | Medium |
| ALL_34 | VDLD (DNR×4) | CAM×2 | HD-MTX 5g/m^2^×4 | VDLD+ CAM | 6-MP+MTX/VD+ intrathecal injection | Medium |
| ALL_35 | VDLD (DNR×4) | CAM×2 | (HR-1'，HR-2'，HR-3') ×2 | VDLD+ CAM | 6-MP+MTX/CA/VD+ intrathecal injection | High |
| ALL_36 | VDLD (DNR×4) | CAM×2 | (HR-1'，HR-2'，HR-3') ×2 | VDLD+ CAM | 6-MP+MTX/CA/VD+ intrathecal injection | High |
| ALL_37 | VDLD (DNR×4) | CAM×2 | (HR-1'，HR-2'，HR-3') ×2 | VDLD+ CAM | 6-MP+MTX/CA/VD+ intrathecal injection | High |
| ALL_38 | VDLD (DNR×4) | CAM×2 | (HR-1'，HR-2'，HR-3') ×2 | VDLD+ CAM | 6-MP+MTX/CA/VD+ intrathecal injection | High |
| ALL_39 | VDLD (DNR×4) | CAM×2 | (HR-1'，HR-2'，HR-3') ×2 | VDLD+ CAM | 6-MP+MTX/CA/VD+ intrathecal injection | High |

*VDLD:* vincristine, daunorubicin, lasparaginase, dexamethasone; *DNR:* daunorubicin; *CAM:* cyclophosphamide, cytarabine, 6-mercaptopurine; *HD-MTX:* high-dose methotrexate; *HR-1'，HR-2'，HR-3'*: high-risk module scheme of BFM collaboration group 1',2',3'; *VDLD* *(Delayed intensive therapy)*: vincristine, adriamycin, lasparaginase, dexamethasone; *6-MP*: 6-mercaptopurine; *HTX/VD*: methotrexaten and/or vincristine, dexamethasone; *MTX/CA/VD*: methotrexaten and/or cyclophosphamide, cytarabine and/or vincristine, dexamethasone.
